# Systematic prediction of DNA shape changes due to CpG methylation explains epigenetic effects on protein–DNA binding

**DOI:** 10.1186/s13072-018-0174-4

**Published:** 2018-02-06

**Authors:** Satyanarayan Rao, Tsu-Pei Chiu, Judith F. Kribelbauer, Richard S. Mann, Harmen J. Bussemaker, Remo Rohs

**Affiliations:** 1 Computational Biology and Bioinformatics Program, Department of Biological Sciences, University of Southern California, Los Angeles, CA 90089 USA; 2 Department of Biological Sciences, Columbia University, New York, NY 10027 USA; 3 Department of Systems Biology, Columbia University, New York, NY 10032 USA; 4 Department of Biochemistry and Molecular Biophysics, Columbia University, New York, NY 10032 USA; 5 Mortimer B. Zuckerman Mind Brain Behavior Institute, Columbia University, New York, NY 10027 USA; 6 Department of Neuroscience, Columbia University, New York, NY 10027 USA; 7 Department of Chemistry, University of Southern California, Los Angeles, CA 90089 USA; 8 Department of Physics & Astronomy, University of Southern California, Los Angeles, CA 90089 USA; 9 Department of Computer Science, University of Southern California, Los Angeles, CA 90089 USA

**Keywords:** *methyl*-DNAshape, 5-methylcytosine, DNA methylation, Epigenetics, DNA structure, DNase I cleavage sensitivity, Human Hox protein binding specificity

## Abstract

**Background:**

DNA shape analysis has demonstrated the potential to reveal structure-based mechanisms of protein–DNA binding. However, information about the influence of chemical modification of DNA is limited. Cytosine methylation, the most frequent modification, represents the addition of a methyl group at the major groove edge of the cytosine base. In mammalian genomes, cytosine methylation most frequently occurs at CpG dinucleotides. In addition to changing the chemical signature of C/G base pairs, cytosine methylation can affect DNA structure. Since the original discovery of DNA methylation, major efforts have been made to understand its effect from a sequence perspective. Compared to unmethylated DNA, however, little structural information is available for methylated DNA, due to the limited number of experimentally determined structures. To achieve a better mechanistic understanding of the effect of CpG methylation on local DNA structure, we developed a high-throughput method, *methyl*-DNAshape, for predicting the effect of cytosine methylation on DNA shape.

**Results:**

Using our new method, we found that CpG methylation significantly altered local DNA shape. Four DNA shape features—helix twist, minor groove width, propeller twist, and roll—were considered in this analysis. Distinct distributions of effect size were observed for different features. Roll and propeller twist were the DNA shape features most strongly affected by CpG methylation with an effect size depending on the local sequence context. Methylation-induced changes in DNA shape were predictive of the measured rate of cleavage by DNase I and suggest a possible mechanism for some of the methylation sensitivities that were recently observed for human Pbx-Hox complexes.

**Conclusions:**

CpG methylation is an important epigenetic mark in the mammalian genome. Understanding its role in protein–DNA recognition can further our knowledge of gene regulation. Our high-throughput *methyl*-DNAshape method can be used to predict the effect of cytosine methylation on DNA shape and its subsequent influence on protein–DNA interactions. This approach overcomes the limited availability of experimental DNA structures that contain 5-methylcytosine.

**Electronic supplementary material:**

The online version of this article (10.1186/s13072-018-0174-4) contains supplementary material, which is available to authorized users.

## Background

Cytosine methylation is the most abundant of all epigenetic marks found on DNA. At the molecular level, cytosine methylation involves the addition of a methyl (CH_3_) group to the C5 atom of cytosine, yielding 5-methylcytosine (5mC). In mammalian genomes, this alteration often occurs in the context of the CpG dinucleotide and is referred to as “CpG methylation” or “DNA methylation.” Ever since 5mC was proposed as a potential epigenetic factor capable of altering gene regulation and cellular differentiation [1], research in this field has been quite active. A recent review [2] highlights the complexity in the interpretation of epigenetic data and the evolution of the definition of epigenetics as the field has advanced.

Although the addition of a single methyl group at the major groove edge leads to only a subtle change in DNA structure, important functional effects have been observed at different scales. For example, methylation-induced alterations in gene expression have been observed in regulatory regions [3–5], and an increase in DNA methylation in one of the X-chromosomes in the female genome can lead to X-chromosome inactivation [6, 7]. Effects of methylation have been studied in two main contexts, genome organization and protein–DNA interactions. Owing to recent advances in technology, DNA methylation profiling can now be performed for any given genome [8–10]. Furthermore, in vitro approaches have recently been used to profile systematically the influence of methylation on DNA binding for human transcription factors (TFs) [11–14], by using variants of universal protein-binding microarray (PBM), high-throughput systematic evolution of ligands by exponential enrichment (HT-SELEX), and SELEX in combination with massively parallel sequencing (SELEX-seq). These approaches revealed that methylation affects binding across the affinity range and that the effect varies within and between TF families [13, 15–17].

To achieve mechanistic insights into these phenomena, detailed understanding of the biophysical and structural effects of DNA methylation is required. Some proteins, such as the Lac repressor, prefer having a bulky methyl group in the major groove and form hydrophobic contacts to this group [18]. By contrast, MspI, a *Moraxella* sp. restriction endonuclease, recognizes the CCGG sequence irrespective of methylation status [18]. These context-dependent effects may be explained in terms of three possible readout mechanisms: direct contacts [19], competitive binding [20, 21], and structural readout [22]. Direct contact to a methyl group allows for the possible formation or alteration of van der Waals interactions, which can either completely abolish or enhance binding [19, 23]. For example, CpG methylation of the cyclic adenosine monophosphate (cAMP) response element half-site (half-CRE) confers binding of CCAAT/enhancer-binding protein alpha (C/EBPα) and C/EBPβ and abolishes binding of CREB, c-Jun, JunD, and ATF2 [24]. In a competitive binding mechanism, the methyl-CpG binding protein (MeCP2) initially binds methylated CpG sites and then blocks sites for other proteins to bind [20, 21]. Many TFs seem to employ one of these first two mechanisms, as revealed by in vitro binding assays [23]. In the case of structure-mediated methylation sensitivity, first demonstrated for the endonuclease DNase I [25], local DNA shape changes enhance binding to target sites already preferred by particular DNA-binding proteins. While direct contacts with the methyl group confer binary effects, the shape-dependent effect is sequence context dependent and can fine-tune the binary direct contact mechanism.

**Fig. 1:**
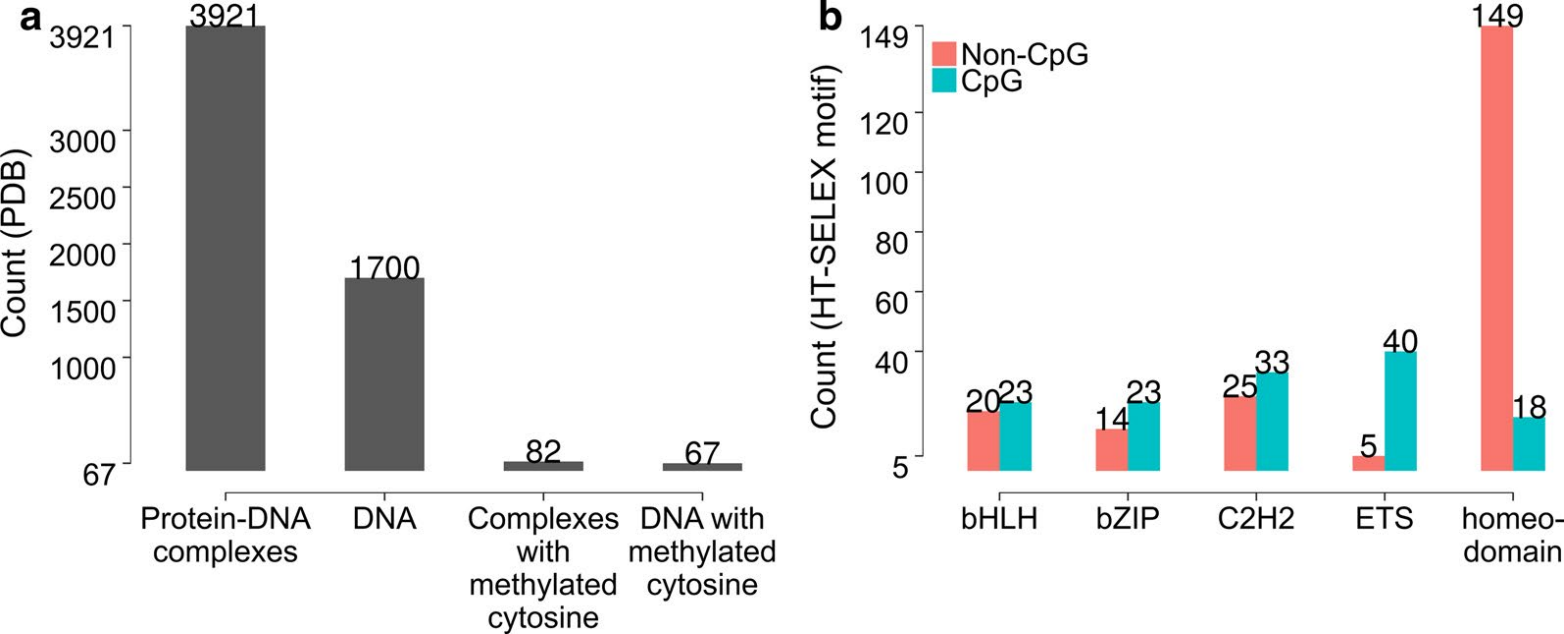
Current statistics of available structures and abundance of CpG dinucleotides in TF binding sites. **a** Count statistics of protein–DNA complex and unbound DNA structures available in the PDB as of 31 May 2017. Counts of subsets of structures (right two bars) containing methylated DNA at CpG site(s) or in other sequence contexts were two orders of magnitude lower than the count of structures containing unmethylated DNA. Systematic profiling of the effect of methylation on three-dimensional DNA structure would require a substantially larger number of structures. Counts include structures solved by X-ray crystallography and NMR spectroscopy. **b** Abundance of CpG steps in TF binding motifs in HT-SELEX data for human TF datasets [29], derived using MotifDb [51]. CpG dinucleotides can be observed in binding sites irrespective of TF family. Five largest human TF families (based on number of binding sites containing at least one CpG step) are specified. Almost 90% of ETS family motifs contain CpG steps. Numbers on each bar represent counts of motifs containing CpG or no CpG steps

Here, we introduce a methodology that enables quantitative probing of the shape-dependent methylation effect. We recently studied how DNA shape contributes to protein–DNA recognition [26–28]. However, we have not yet systematically quantified the effect of DNA methylation on protein binding [22]. Motivated by the widespread occurrence of CpG dinucleotides in TF binding motifs of different protein families [29–31], we aimed to study CpG methylation in the context of gene regulation (Fig. 1b). Understanding the protein–DNA readout of methylated cytosine requires structural insight derived from experimentally determined structures. Unfortunately, the current content of the Protein Data Bank (PDB) [32] includes only a few structures containing cytosine modifications (Fig. 1a). To close this knowledge gap, we utilized computational modeling of many DNA fragments to study the intrinsic effects induced by cytosine methylation, in a manner analogous to previous high-throughput studies of DNA shape of unmethylated genomic regions [33–35]. The resulting query tables can be utilized to analyze systematically the effect of methylation on protein–DNA interactions, as we demonstrate for DNase I cleavage and Pbx-Hox binding data.

## Methods

### Sequence and structure datasets

A total of 3518 DNA fragments of lengths varying from 13 to 24 base pairs (bp) were considered in all-atom Monte Carlo (MC) simulations, based on a previously published protocol (see Additional file 1 for details) [36]. Before performing simulations, we added 5-methyl groups at CpG steps to the core sequence (central regions in sequences in Additional file 2: Table S1) of every DNA fragment [25]. Sequences of these fragments were designed to capture the complete pentamer space in terms of the sequence context. Each considered sequence was defined as having at least one CpG step. For better coverage of the sequence space, four different nucleotide combinations were used to flank each designed sequence. Canonical B-DNA structures for all DNA fragments were generated by the JUMNA program [37] and used as input for the all-atom MC simulations [36].

### All-atom MC simulations

**Fig. 2:**
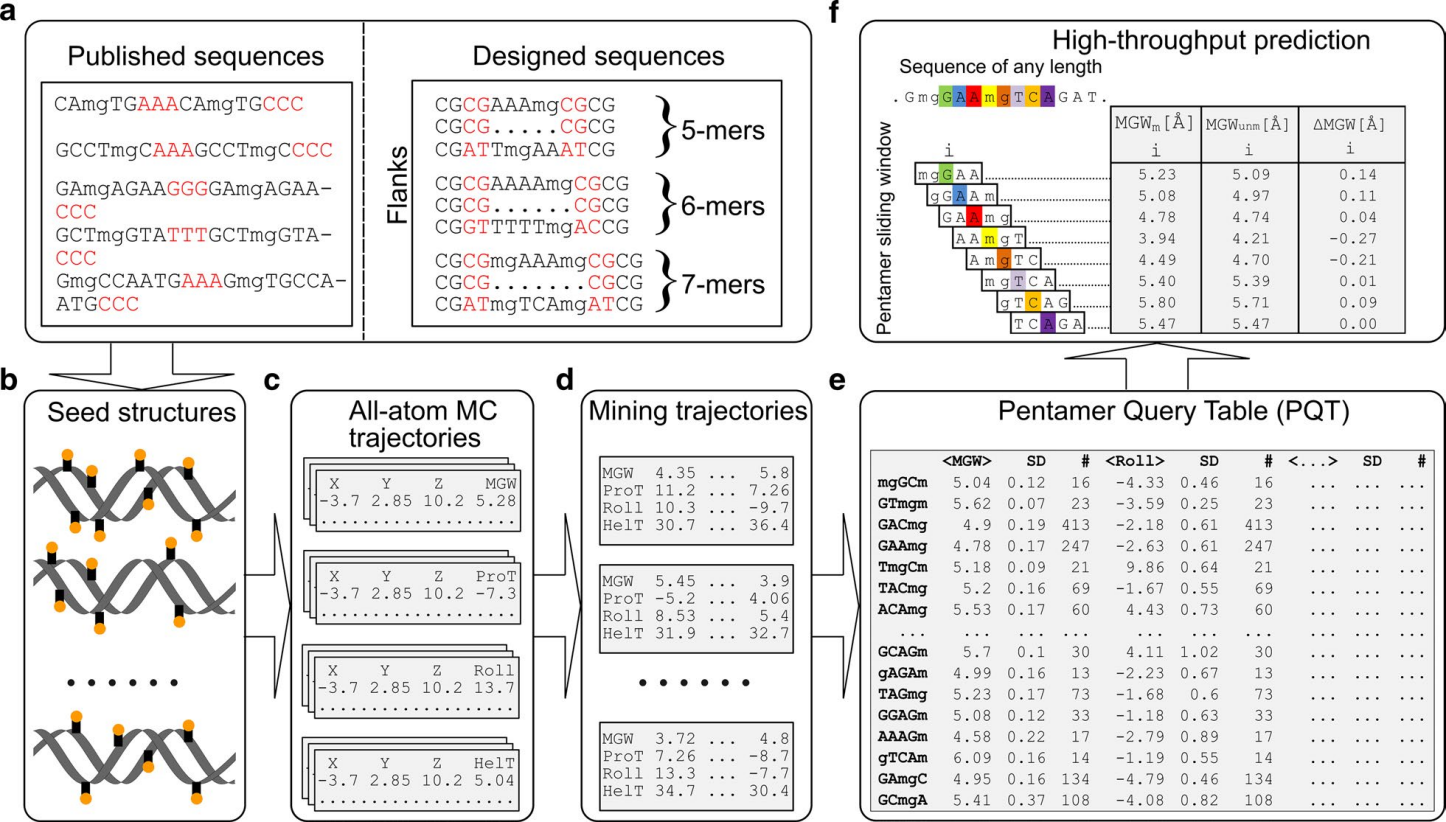
Workflow for high-throughput *methyl*-DNAshape method. **a** Sequence pool. DNA fragments were considered for MC simulations to capture a sequence space that includes CpG methylation. Published sequences (left rectangular box) [23] and manually designed sequences (right rectangular box) included DNA fragments comprising a variable core (containing at least one methylated CpG step, called “mg” step) and flanks (4 bp in length). Right flanks were reverse complements of left flanks. For a given length of core sequence (5, 6, or 7 bp), all possible sequences (Additional file 1) were considered for MC simulations. **b** Seed structures. Canonical B-DNA structures were generated for all selected sequences. The 5-methyl groups (orange circles) were introduced at cytosine positions with letter “m” (on Watson and Crick strand). **c** All-atom MC trajectories. Simulations were performed on seed structures for 2 million MC cycles, with snapshots recorded every 100 cycles after equilibration. **d** Mining trajectories. Recorded snapshots were analyzed for DNA shape features (see Additional file 1: Supplementary methods) associated with corresponding DNA sequences. **e** Pentamer Query Table (PQT). Pentamer sliding-window approach was applied to analyzed DNA fragments. Calculated DNA shape features (HelT, MGW, ProT, and Roll) were recorded at the center of each pentamer. Assigned value for a corresponding shape feature represents the average of all shape feature values in the sequence pool for a given pentamer in the PQT. **f** Front-end interface. Our easy-to-use *methyl*-DNAshape web server or DNAshapeR Bioconductor/R package can be used to profile shape features of any genomic region and DNA sequences of any length by using a pentamer sliding-window approach. The *methyl*-DNAshape web server, available at http://rohslab.usc.edu/methyl-DNAshape/, also outputs the effect of methylation on shape features in terms of Δshape (shown here for MGW)

MC simulations (Fig. 2c) traverse the energy landscape by making random moves [38], thus combining effective sampling with fast equilibration [39]. For this study, MC sampling was expanded to include 5mC. Rotation of the 5-methyl group added one degree of freedom, whose rotation was implemented in a manner analogous to that of the thymine 5-methyl group. Partial charges for 5mC were taken from a database of AMBER force fields for naturally occurring modified nucleotides [25, 40]. For a given DNA structure, the MC simulation protocol included two million MC cycles, with each cycle attempting random variations of all degrees of freedom (Additional file 3: Table S2). After completion of the MC simulations, trajectories were analyzed by using snapshots that were stored every 100 MC cycles. After we discarded the first half-million MC cycles as an equilibration period, we mined the remaining trajectories using CURVES analysis [41] (Fig. 2d; see Additional file 1 for detailed description of methodology).

### Building the *methyl*-DNAshape Pentamer Query Table

Mining of the MC trajectories generates average structural features for a given sequence. We assigned minor groove width (MGW) values to nucleotides in a strand-independent manner [33]. We adopted a pentamer sliding-window approach to record DNA shape feature values from representative structures. For a given sequence of length *N*, the approach profiled the shape features of *N* − 4 pentamers due to end effects. For MGW and propeller twist (ProT), values were assigned to the central bp of the corresponding pentamer. For Roll and helix twist (HelT), two values were recorded for bp steps 2–3 and 3–4 of a pentamer, respectively. Shape feature values from multiple occurrences of a given pentamer in different DNA fragments were averaged and assigned as representative values for that pentamer (Additional file 4: Fig. S1).

All possible pentamers were categorized in unmethylated and methylated groups. Unmethylated pentamers contained letters from the standard DNA alphabet, {A, C, G, T}. Methylated pentamers contained letters from the expanded alphabet, {A, C, G, T, m, g}. We assigned the letter “m” to 5mC and lowercase “g” to guanine base-paired with 5mC. We considered there to be no partial methylation; thus, for a DNA fragment of length *N*, methylation on the forward strand at index *i* (5′–3′) also indicates methylation at index *i* + 1 (3′–5′) on the reverse strand. The G base-paired to 5mC in a methylated 5mC/G bp cannot be treated in a similar fashion as G base-paired to unmethylated C. In addition, due to the requirement of DNA methylation at both Cs of a CpG step, each 5mC will be followed by a G base-paired to another 5mC on the opposite strand. Thus, “m” and “g” cannot be considered as independent letters.

Introduction of the two letters “m/g” for a 5mC/G bp increased the number of possible unique pentamers, with 475 new pentamers being added to the 512 unique pentamers representing unmethylated DNA (Additional file 5: Table S3). Here, we discuss two specific examples. In the first example, NNmgN where N ∈ {A, C, G, T} has a single methylation mark at the underlined position 3. The second example is the complex case of gmgNm. To assign shape feature values, we have to consider that 5mC precedes “g” on its 5′ flank and that “g” follows “m” on its 3′ flank (Additional file 6: Fig. S2). We ran MC simulations with these combinations of methylated CpG steps to enrich pentamers of these types of compositions (see Additional file 7 for list of all sequences studied with MC simulations).

### *methyl*-DNAshape method for high-throughput prediction of methylated DNA shape features

The *methyl*-DNAshape method derives DNA shape features of methylated DNA at nucleotide resolution, while considering the local sequence context. In a manner analogous to our DNAshape method for unmethylated DNA [33], we used a pentamer centered at position *i* to estimate DNA shape features at that position. We adopted the equivalent approach for DNA with methylated CpG dinucleotides, to capture the methylation properties of mammalian genomes. We derived the *methyl*-DNAshape Pentamer Query Table (*m*PQT), in analogy to the DNAshape Pentamer Query Table (PQT). DNA shape features at nucleotide position *i* were determined by querying the *m*PQT based on a pentamer using two neighboring nucleotides in both flanks (P_*i*_ = N_*i*-2_N_*i*-1_N_*i*_N_*i*+1_N_*i*+2_). Ultimately, *methyl*-DNAshape calculates four feature vectors, one for each of the shape features HelT, MGW, ProT, and Roll (Fig. 2).

As in our previous work, we selected four DNA shape features that play important roles in protein–DNA recognition [33]. ProT is an intra-bp parameter that accounts for bp twisting along the base-pairing axis. Increased values of ProT lead to an opportunity to form an additional inter-bp hydrogen bond in the major groove [28]. Roll and HelT are bp step features that estimate deformation at the dinucleotide level. The MGW feature plays a pivotal role in DNA shape readout [27]. A narrow minor groove enhances negative electrostatic potential and offers favorable interactions for positively charged amino acids [27]. Although the scarcity of experimentally solved structures with CpG methylation prohibited us from performing a validation such as is possible for unmethylated structures, we compared MGW predictions using *methyl*-DNAshape with X-ray co-crystal structures (Additional file 8: Fig. S3). The *methyl*-DNAshape method is available as a web server at http://rohslab.usc.edu/methyl-DNAshape/ and as an extension to the R/Bioconductor package DNAshapeR [42] at http://bioconductor.org/packages/devel/bioc/html/DNAshapeR.html.

## Results and discussion

### Effect of CpG methylation on DNA shape features

To quantify the effects of cytosine methylation on DNA shape features, we compared values for all unique pentamers that contained a single CpG step, as derived from DNAshape [33] (designed for unmethylated DNA) and *methyl*-DNAshape (our high-throughput prediction method designed for methylated DNA; see “Methods” section). We considered four DNA shape features—HelT, MGW, ProT, and Roll—in this analysis.

**Fig. 3:**
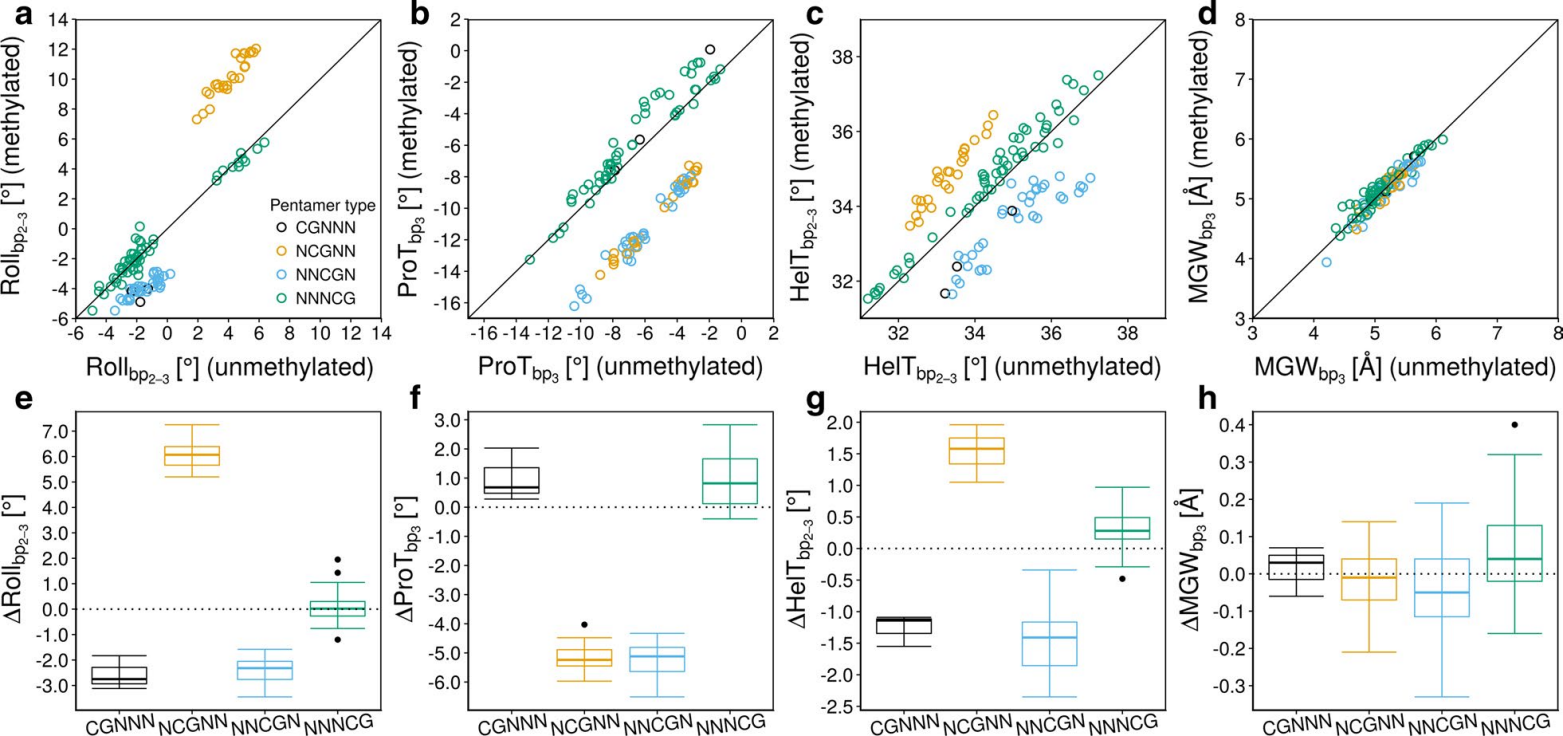
Effect size of CpG methylation on DNA shape features. Methylation-induced changes were analyzed for four shape features: **a**, **e** roll, **b**, **f** propeller twist (ProT), **c**, **g** helix twist (HelT), **d**, **h** minor groove width (MGW). For each shape feature, values for pentamers from the DNAshape query table for unmethylated DNA were plotted against values for corresponding pentamers from the *methyl*-DNAshape query table for methylated DNA. For simplicity, pentamers with one and only one CpG/mpg step (where “m” represents 5-methylcytosine and “g” represents G base-paired with “m” on the reverse-complement strand) were considered, for a total of 116 occurrences (Additional file 1). For bp step features Roll and HelT, values at bp steps 2–3 of each pentamer were used. For MGW and ProT, values at the central bp of each pentamer were used. CpG methylation increased Roll by an order of magnitude (light-orange dots). The opposite was observed when methylation occurred at the immediate next bp step (light-blue dots). Presence of a methyl group at the central bp, either on the forward (light-blue dots) or reverse (light-orange dots) strand caused a decrease in ProT

Roll and ProT exhibited strong methylation effects (50–100% of the range observed across all unmethylated-DNA sequences). At methylated CpG steps, Roll increased by an average of 6° (range 5.1°–7.2°), representing a similar effect size as previously observed in molecular dynamics simulations [43]. In methylated C/G bp, ProT decreased by an average of 5° (range − 4.5° to − 6.0°). By contrast, we observed relatively small effects for MGW and HelT (Fig. 3). An increase in Roll caused partial unstacking of the bp step, leading to widening of the minor groove. This conformational change might affect hydrogen bond formation in the major groove by exposing amino groups of guanine bases and oxygens of cytosine bases with different relative orientations. Presence of a methylated CpG step at position 1 or 3 (in the 5′–3′ direction) in pentamers resulted in a lowering of HelT by ~ 2° (Fig. 3c). Only subtle changes in MGW were observed, except for some particular sequence contexts.

### Effect of CpG methylation on MGW of A-tracts

A-tracts, or poly[A/T] tracts, consist of a continuous run of at least three As or Ts without any TpA step. A-tracts, which play an important role in TF-DNA binding [44, 45], have a rigid conformation due to inter-bp hydrogen bonds in the major groove [46].

**Fig. 4:**
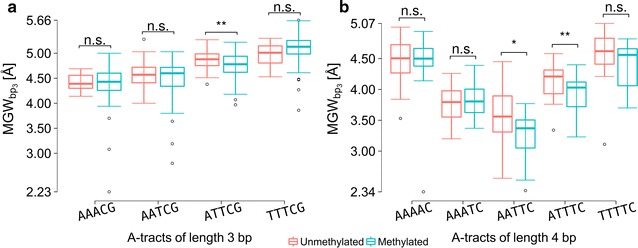
Effect of CpG methylation on minor groove width (MGW) of adjacent A-tracts. **a** MGW values at the central nucleotide of 3-bp A-tracts, which are shown from AAACG to TTTCG with an exchange of one bp (A/T to T/A) from the 3′ end. Methylation did not decrease MGW at the central bp, except in the ATTCG sequence. Wilcoxon test *P* values were calculated for methylation narrowing the minor groove at the central nucleotide as the alternative hypothesis (*0.01 < *P* value ≤ 0.05; **0.001 < *P* value ≤ 0.01). Four A-tracts followed by a CpG step at the 3′ end include A-tracts preceded by a CpG step at the 5′ end because of symmetry in sequence and cytosine methylation. **b** MGW at the central nucleotide of 4-bp A-tracts follows a bell-shaped curve from AAAAC to TTTTC. One bp at a time was exchanged from A/T to T/A, starting at the 3′ end. Paired *t* test *P* values were calculated for methylation narrowing the minor groove at the central bp as the alternative hypothesis. Two pentamers, AATTC and ATTTC, showed significant *P* values, meaning that methylation narrowed the minor groove. MC simulations were performed on longer DNA fragments containing hexamer sequences with a CpG/mpg bp step at position 5, and MGW values were measured at the central position 3

We analyzed the effect of methylation on the MGW of A-tracts flanked by CpG steps. As we derived the shape features from pentamers, we considered A-tracts of limited length of either three (e.g., AAACG; Fig. 4a) or four (e.g., AAAAC; Fig. 4b) nucleotides. For A-tracts that were three bp in length, the subsequent CpG context extended into one nucleotide position flanking the pentamer because 5mC at the fifth position of a pentamer implicitly assumes a G/5mC bp at the following position. Box plot analysis revealed that the observed narrowing or widening of the minor groove upon CpG methylation depended on the sequence composition of As and Ts in the A-tract. For example, consecutive mutation from A to T in AAAAC led to a bell-shaped MGW profile, due to the introduction of a flexible TpA “hinge” step [47]. Maximal narrowing of the minor groove upon CpG methylation was observed for AATTC (Fig. 4b). This result might be due to the fact that this particular A-tract had a narrow minor groove, an effect that was amplified through cytosine methylation in the adjacent CpG step. Effects of DNA methylation on MGW were larger and more variable for 4-bp than for 3-bp A-tracts. This result was likely due to the more distinct minor groove narrowing of longer A-tracts and suggests that the methylation effect can be amplified depending on the A-tract features of the surrounding sequence.

Bulky methyl groups introduced by CpG methylation subtly widened the major groove and, in turn, narrowed the minor groove [22]. This observation can be explained in part by the proximity to the phosphate backbone of the methyl group of 5mC [22]. Narrowing of the minor groove enhances the negative electrostatic potential and, thereby, attracts minor groove-binding basic side chains more efficiently [22, 25]. This mechanism could potentially be employed when A-tracts reside in vicinity of CpG dinucleotides, as previously reported for various methyl group-binding proteins that use arginine-carrying AT-hooks [48] to recognize A-tracts adjacent to a CpG-containing motif [11].

### Application of *methyl*-DNAshape predictions: modeling of DNase I cleavage activity

**Fig. 5:**
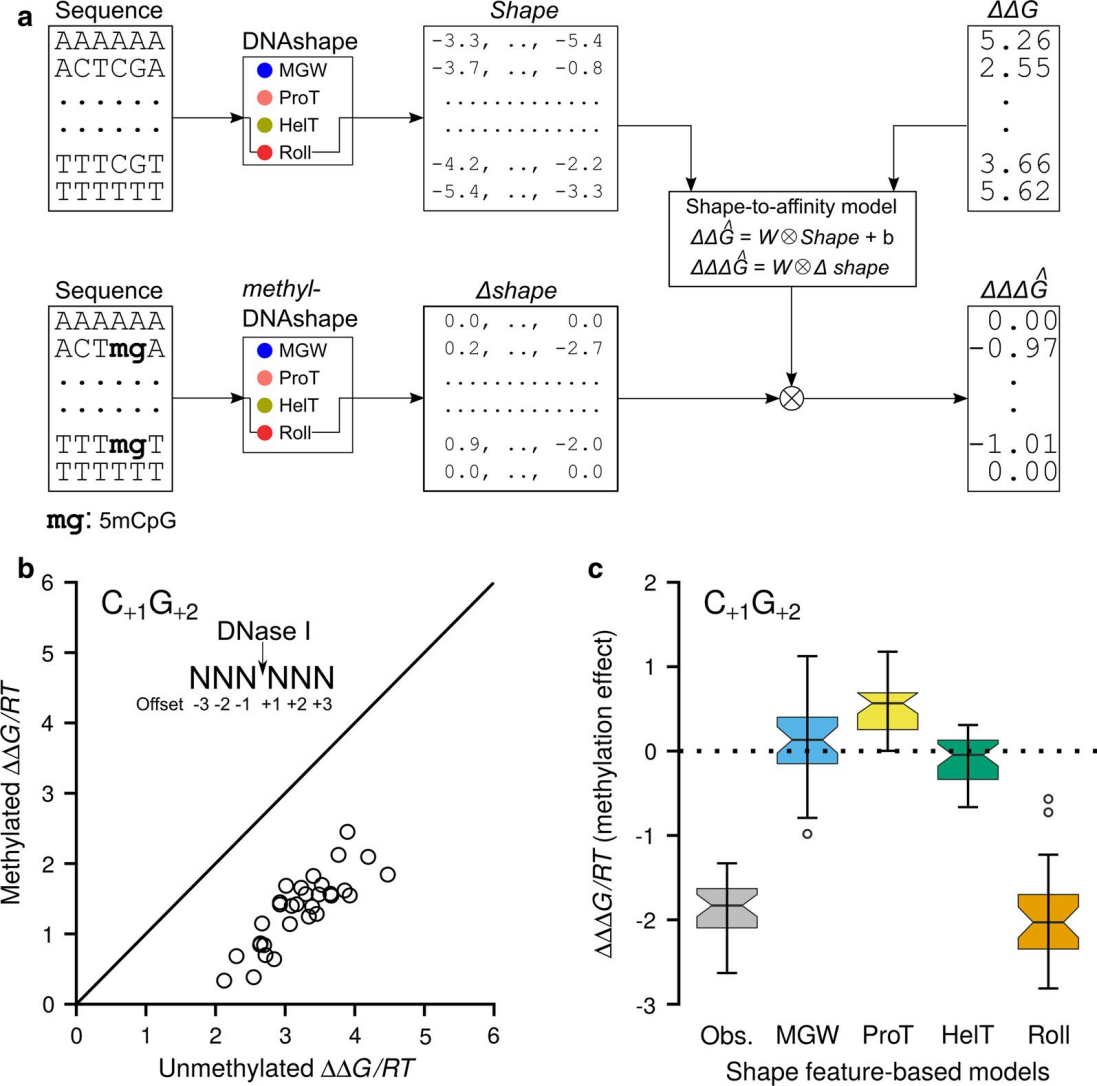
Modeling of methylation-induced shifts in cleavage rates using methylation-induced shifts in shape feature profile. **a** Points on plot represent inferred binding free energy (ΔΔ*G/*RT) values of DNase I to unmethylated hexamers and corresponding methylated hexamers with absolute phosphate cleavage count ≥ 25. Methylation-induced effects are shown for sequences with C_+1_G_+2_ offset. Shift (downward) from diagonal indicates log-fold increase in cleavage activity of DNase I for methylated hexamers. **b** Shape-to-affinity modeling and use of *methyl*-DNAshape features. Shape-to-affinity model (L1- and L2-regularized linear regression model) built using unmethylated data. DNA shape features for unmethylated hexamers and their corresponding free energies (ΔΔ*G/*RT) were used as predictors and response variables, respectively. The model used the methylation effects on shape features (Δshape) calculated by *methyl*-DNAshape to predict ΔΔΔ*G* (methylation effects on free energy, indicated by ΔΔΔ*Ĝ*). Linearity of the model allowed direct use of Δshape as input variable. Roll values are shown for illustration purposes. **c** Predictive powers of different shape-based models. Observed ΔΔΔ*G/*RT with median around − 2 is shown in gray colored box. Roll-based model accurately predicts the cleavage bias for C_+1_G_+2_ offset

The DNA shape-dependent mechanism by which DNase I cleaves naked genomic DNA [22] serves as appropriate test system for assessing the functional relevance of our predictions of methylation-induced shape changes. In particular, the hexamer-based model (3-bp up- or downstream of the phosphate cleavage site) explained most of the variance in cleavage rates (Additional file 9: Table S4; Additional file 10: Table S5). Enhanced cleavage by DNase I was observed for hexamers containing a CpG step at the + 1/+ 2 positions (referred to as C_+1_G_+2_ or positions 4 and 5 in a hexamer from the 5′ direction) immediately adjacent to the central cleavage site (Fig. 5a).

To assess how methylation-induced shape changes relate to the binding free energy (ΔΔ*G/*RT) of DNase I, we developed shape-based statistical models for unmethylated DNA (Fig. 5b). We used hexamers with an observed cleavage count of at least 25 to build our predictive models (Additional file 1). Next, we evaluated how well the resulting linear model predicted the effect of methylation on DNase I binding/cleavage (ΔΔΔ*G/*RT = ΔΔ*G/*RT_methylated_ − ΔΔ*G/*RT_unmethylated_) in terms of the effect of methylation on shape (Δshape = shape_methylated_ − shape_unmethylated_) (Additional file 1).

To evaluate the predictive power of each individual shape feature, we trained models based on each shape feature category and plotted the predicted ΔΔ*G* shift against the maximum observed ΔΔ*G* shift for a C_+1_G_+2_ offset (Fig. 5c). The Roll-based model better explained the shift than models based on other shape features. This observation may reflect the causal effect of the influence of methylation on DNA shape features (Fig. 3).

We observed an enhanced negative value (− 0.187) at the + 1/+ 2 offset in the weight vector ***W*** (Fig. 5b) of the Roll-based model. This finding suggested that the methylation-induced increase in Roll at this CpG offset caused a decrease in ΔΔ*G* and, thus, an increase in binding affinity. For the C_+1_G_+2_ offset, the observed ΔΔ*G* shift was well predicted by the change in Roll (Fig. 5c and Additional file 1). Compared to earlier work that was limited to MC simulations of a restricted set of methylated-DNA fragments [25], the *methyl*-DNAshape approach presented here enables systematic probing of the methylation effect for any CpG offset, number of sequences, or entire genomes.

### CpG methylation effects on DNA binding of human Pbx-Hox complexes

**Fig. 6:**
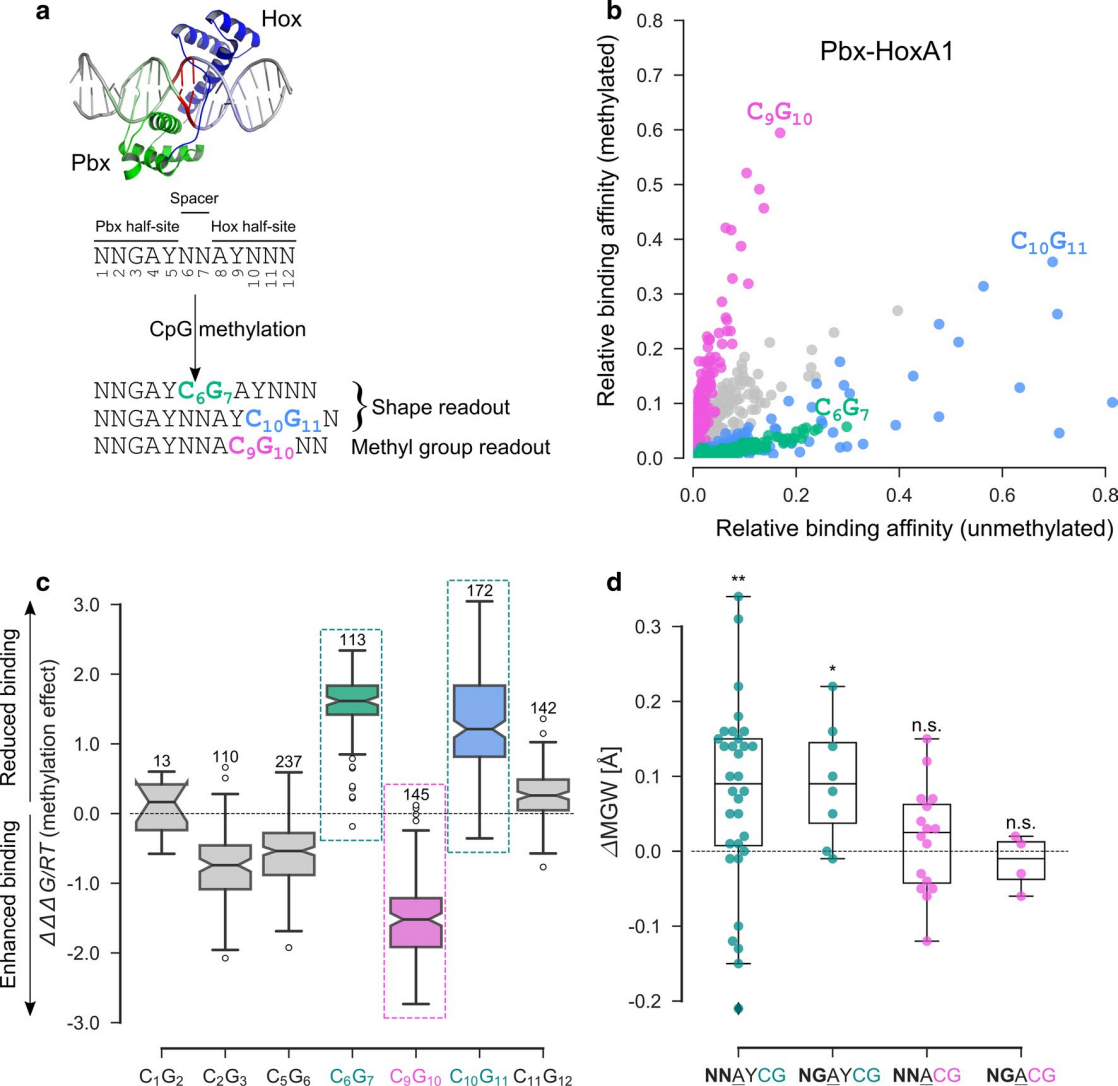
CpG methylation induces a DNA shape change that explains its effect on Pbx-Hox binding. **a** Schematic representation of Pbx-Hox heterodimer bound to DNA (PDB ID 1PUF), and of the effect of CpG methylation on binding. Pbx (green) and Hox (blue) homeodomains bind up- and downstream of the central spacer region (indicated in red), respectively. CpG methylation at offsets 6/7 and 10/11 reduces binding, whereas methylation at offset 9/10 enhances binding. Methyl group readout was previously identified as underlying mechanism for the latter offset [13]. **b** Scatter-plot representation of relative binding affinities of methylated versus unmethylated sequences for Pbx-HoxA1 complex. Sequences carrying a single methylation event and their corresponding unmethylated part were considered. Green, magenta, and blue points correspond to methylation at offsets 6/7, 9/10, and 10/11, respectively. Sequences containing CpG dinucleotides at other offsets (relatively weakly affected by methylation) are colored gray. **c** Alternative representation of the data in **b**, showing the effect of methylation on binding free energy, denoted as ΔΔΔ*G/*RT. Positive (e.g., offsets 6/7 and 10/11) and negative (e.g., offset 9/10) shifts from the dashed line (indicating no methylation effect) reflect reduced and enhanced binding (on logarithmic scale) due to methylation. CpG dinucleotides at offsets 6/7 and 10/11 produce the same hexamer context for A_4_ and A_8_ (NNAYCG/NGAYCG) and, hence, were assigned a common color, dark-cyan. **d** Analysis of the methylation-induced change in MGW at positions A_4_ and A_8_ within the Pbx-Hox binding site (NNGAYNNAYNNN), for the different hexameric/pentameric contexts that the Pbx**-**Hox heterodimer may encounter within its binding sequence. Coloring corresponds to that of labels and rectangular patches in **c**. Statistically significant widening of minor groove (first two boxes) plausibly explains the observed reduced binding due to methylation at CpG offsets 6/7 and 10/11. No significant change in MGW upon methylation was observed for offset 9/10

In previous reports, SELEX-seq profiling followed by DNA shape analyses of binding by heterodimers of all eight *Drosophila melanogaster* Hox proteins in complex with their common co-factor Extradenticle (Exd) revealed an important role for MGW readout [26, 49]. More recently, an extension of the SELEX-seq method for methylated binding sites, EpiSELEX-seq, revealed that cytosine methylation modulates the affinity with which human orthologs (Pbx-Hox) of these heterodimers bind to CpG dinucleotide-containing sites [13]. The DNA sequences associated with the largest binding affinity for the Exd-Hox and Pbx-Hox complexes matched the 12-bp sequence pattern NTGAYNNAYNNN, where Y represents pyrimidine (C or T) and N any nucleotide (Fig. 6a).

As previously reported [13], direct comparison of the relative binding affinities for unmethylated versus methylated sequences (Fig. 6b, c) shows that cytosine methylation can either have a stabilizing or destabilizing effect on Pbx-Hox binding, depending on the position of the CpG dinucleotide within the binding site. For example, methylation of a CpG dinucleotide at offset 6/7 (NTGAYCGAYNNN; C_6_G_7_; green points/box in Fig. 6b, c) and offset 10/11 (NTGAYNNAYCGN; C_10_G_11_; blue points/box in Fig. 6b, c) suppresses binding, whereas methylation at offset 9/10 (NTGAYNNACGNN; C_9_G_10_; magenta points/box in Fig. 6b, c) enhances binding by an order of magnitude. We previously proposed a plausible mechanism for the latter stabilizing effect, which we postulated to involve direct contacts to the methyl group in the major groove [13]. However, an explanation of the suppressed binding at the CpG offsets 6/7 and 10/11 was lacking (Fig. 6a).

No protein–DNA contact was observed in the co-crystal structure (PDB ID: 1PUF) [50] at offset 6/7. However, the nucleotides at offset 6/7 form a spacer located between two AY dinucleotides (Fig. 6a), which were previously shown to exhibit strong shape preferences. Specifically, minor groove narrowing at AY positions adjacent to the central spacer was shown to be associated with enhanced binding when the nucleotide sequence was varied for unmethylated DNA [26, 49]. Therefore, we hypothesized that a methylation-induced change in DNA shape near the CpG dinucleotide could affect binding affinity. We used the pentamer-based shape tables that form the foundation of DNAshape [33] and *methyl*-DNAshape to investigate this effect systematically.

A pentamer window centered at the A_8_ position includes a CpG dinucleotide at offset 9/10 within its 5 bp (NNGAYNNA**CG**NN). However, a CpG step at offsets 6/7 and 10/11 only includes one bp of the CpG dinucleotide (NNGAY**CG**AYNNN or NNGAYNNAY**CG**N) and indirectly constrains the nucleotide identity at a sixth position after the pentamer window. This distinction became important when we predicted MGW. In the case of the methylated-DNA table (*m*PQT), the presence of a (methylated) C at position 5 within the pentamer implies the presence of a G at the following position in the training set from which the pentamer tables were derived. This prediction is not the case for the unmethylated-DNA table (PQT). The pentamer tables do not capture a weak dependency of shape on the sixth position, which confounds our estimate of the methylation effect on shape. For this reason, we compiled an additional table consisting of unmethylated-DNA shape parameters for all hexamers ending with CpG and heptamers with CpG flanks (Additional file 1), which we used to estimate the effect of methylation on shape. Figure 6d shows that cytosine methylation in a sequence context consistent with the presence of a CpG step at offset 6/7 or 10/11 within the 12-bp Pbx-Hox binding site results in widening of the minor groove (see Additional file 1 for details on statistical tests performed). This observation, combined with the known inverse relationship between MGW and binding affinity for unmethylated DNA, provides a plausible explanation for the methylation-induced weakening of binding observed at these offsets (Fig. 6b). In contrast, no effect of methylation on MGW can be observed for the CpG offset 9/10, where direct contacts in the major groove already provided a mechanistic explanation [13].

## Conclusions

Mechanisms of protein–DNA recognition remain incompletely understood. This lack of knowledge is particularly true for the readout of methylated DNA [15], despite its important role in gene regulation [22]. DNA sequence and shape readout are key factors in achieving TF binding specificity. For base readout, presence of a bulky hydrophobic methyl group in the major groove may facilitate hydrophobic contacts with protein side chains [17]. For shape readout, local structural changes of the double helix induced by cytosine methylation may strengthen or weaken protein contacts to DNA [25]. Here, we describe an approach to probe and comprehend the shape readout mechanism of methylated DNA. As a high-throughput approach for predicting shape features of methylated DNA, our *methyl*-DNAshape method can be used to determine how the intrinsic shape of chemically modified DNA mediates recognition by TFs. Moreover, this method overcomes the limitation of the unavailability of experimental structures containing methylated cytosine.

One possible application of our method is to utilize high-throughput predictions of DNA shape features in quantitative models of protein–DNA binding. We found that the predicted change in shape features due to methylation partially explained the magnitude and context dependence of the experimentally measured effect of CpG methylation on DNase I cleavage [25]. Moreover, we were able to explain previously unexplained effects of DNA methylation on the binding specificity of human Pbx-Hox complexes. This study, therefore, represents a step forward toward a full mechanistic understanding of gene expression regulation.

## Additional files

**Additional file 1: Supplementary methods.** Methodology for structure comparison, MC simulations, pentamer model, DNase I cleavage analysis, and statistical tests.

**Additional file 2. Table S1.** Types of DNA fragments and their counts considered in MC simulations.

**Additional file 3: Table S2.** Variables considered in MC simulations.

**Additional file 4: Figure S1.** Shape vector calculation.

**Additional file 5: Table S3.** Count breakdown of unique pentamer entries in *methyl*-DNAshape Pentamer Query Table (*m*PQT).

**Additional file 6: Figure S2.** Use of CpG context table in ΔMGW prediction.

**Additional file 7.** Sequence pool used in all-atom MC simulations.

**Additional file 8: Figure S3.** MGW profiles for selected DNA fragments or protein–DNA complexes.

**Additional file 9: Table S4.** Data preprocessing of DNase I cleavage data.

**Additional file 10: Table S5.** DNase I cleavage data in hexamer context.
