# Supplementary material for: Systematic prediction of DNA shape changes due to CpG methylation explains epigenetic effects on protein–DNA binding
Source: Epigenetics Chromatin. 2018 Feb 6;11:6. doi: 10.1186/s13072-018-0174-4 (PMC5800008; doi:10.1186/s13072-018-0174-4)
Supplement: Supplementary file 1 — Additional file 1: Supplementary methods. Methodology for structure comparison, MC simulations, pentamer model, DNase I cleavage analysis, and statistical tests. [file 13072_2018_174_MOESM1_ESM.pdf]

SUPPLEMENTARY METHODS

Retrieval of experimentally determined structures from Protein Data Bank (PDB)

To generate count statistics, we used an advanced search interface to query the PDB [1] for occurrences of methylated cytosine. **Fig. 1a** presents the numbers of structures retrieved from the PDB on 31 May 2017 (as a snapshot in time). Counts are expected to evolve over time as new structures are added to the database. Numbers can be updated by running the Python script *QueryPDBCounts.py* (available at https://doi.org/10.5281/zenodo.834334).

PDB IDs of methylated DNA structures

Our analysis revealed a very small subset of structures with methylated CpG dinucleotide step(s). PDB IDs of structures containing methylated CpG step(s) are the following: 1IG4, 1IH3, 1R3Z, 265D, 270D, 2KY8, 2MOE, 329D, 3C2I, 3VXX, 4C63, 4F6N, 4GJP, 4GLG, 4HP1, 4LG7, 4M9E, 4MKW, 4R2A, and 4LT5.

Count statistics of transcription factor (TF) binding motifs containing CpG step(s)

Counts of binding motifs containing CpG step(s) for TF families were retrieved from MotifDb [2], an R package comprising TFBS databases, such as HT-SELEX sequences from Jolma et al. [3], the expanded HT-SELEX dataset published in Yang et al. [4], or JASPAR_CORE [5], TRANSFAC [6], and others.

Types and counts of sequences considered for Monte Carlo (MC) simulations

An ensemble of sequences was selected to represent pentamers with methylated cytosine(s) in the CpG context. Additional file 7 (*sequence_pool.xlsx*) contains selected sequences/fragments that were considered for MC simulations. **Table S1** (Additional file 2) summarizes the counts for different types of fragments.

All-atom Monte Carlo simulations

MC simulations utilize a random sampling method to probe the search space while considering all of the atoms. This method treats most bond angles and all bond lengths as constants, which results in a substantial reduction in the number of degrees of freedom [7]. Variables considered in the MC simulations are summarized in **Table S2** (Additional file 3). MC simulations were performed by using an implicit solvent with sigmoidal distance-dependent dielectric function, explicit sodium counter ions, and associated Jacobians [8].

Total system energy was calculated by using the AMBER force field [9]. The force field for 5mC differed from that of the cytosine parameters due to the added methyl group. We used partial charges derived for 5mC from the database of AMBER force fields for naturally occurring modified nucleotides [10].

MC simulations started from a seed structure (in this case, a canonical B-DNA structure of a sequence generated with standard structural parameters using JUMNA [11]) as input and ran for 2 million MC cycles. Trajectory snapshots were stored every tenth cycle. The first half-million MC cycles were discarded as the equilibration period. Following equilibration, a total of 150,000 snapshots representing 1.5 million MC cycles were stored in the trajectory file of each individual simulation. The trajectory analysis program traversed through all of these snapshots and recorded the average shape parameter values derived by CURVES [12]. The program also generated an average MC structure as a representation of the sequence.

Pentamers of different sequence composition and methylation status

Introduction of the letters "m" for 5-methylcytosine (5mC) and "g" for guanine base-paired to 5mC resulted in a total of 1,974 pentamers. As we considered DNA shape features of a pentamer on the forward strand, this experimental design also covered features of its reverse complement. Thus, the total number of entries in our methylated Pentamer Query Table (*m*PQT) was half of the total count (987). We refer to these 987 pentamers as the unique pentamers for the *methyl*-DNAshape method. Of these, 512 unique pentamers were comprised of the nucleotides A, C, G, and T. The remaining 475 unique pentamers contained at least one of the two newly introduced letters, "m" and "g". **Table S3** (Additional file 5) gives detailed representations of pentamers found in the *m*PQT.

Pentamers used in scatter plot analysis

To understand the influence of a single methylation event on DNA shape features, we considered pentamers with only a single CpG/mpg bp step (**Fig. 3**). With this constraint, a total of 116 (see below) pentamers were selected.

#Pentamers of type 5′-CGNNN-3′ = 64 (covers 5′-NNNCG-3′) … (a)
#Pentamers of type 5′-NCGNN-3′ = 64 (covers 5′-NNCGN-3′) … (b)

Symmetry occurs in eq. (a) for CGNCG, only counts 2 pentamers (CGACG and CGCCG) of this type and redundancy occurs for the count of pentamer CGCGN resulting a total count of 122 pentamers containing at least one CpG step:

$$64 + 64 - 2 \text{ (symmetric)} - 4 \text{ (redundant)} = 122$$

Count of pentamers containing exactly two CpG steps:

#Pentamers of type 5′-CGCGN-3′ = 4
#Pentamers of type 5′-CGNCG-3′ = 2

Total pentamers containing exact one CpG step = 122 – 6 = 116

Illustration of shape vector calculation

We illustrated graphically how the bp step feature values of inter-bp shape features were assigned at each nucleotide position (Additional file 4: **Fig. S1**).

Effect of CpG methylation on minor groove width (MGW) of A-tracts

A-tracts are important for protein-DNA recognition by many TF families [13]. A-tracts are runs of at least three As and Ts without a TpA step characterized by a narrow minor groove, which attracts basic amino acids due to its enhanced negative electrostatic potential [14]. In this study, we explored the effect of DNA methylation on the minor groove geometry of A-tracts. We analyzed the MGW of A-tracts measuring 3 bp or 4 bp in length, followed or preceded by a CpG base-pair (bp) step with methylated cytosine(s). For 3-bp A-tracts, we conducted a nonparametric significance test. Data and procedures to calculate *P*-values in this experiment are detailed below.

We collected data under the alternate hypothesis that CpG methylation narrows the minor groove at the central base pair (third position from 5′ direction) of a pentamer. For 3-bp A-tracts, or poly[$(A/T)_3$], we collected the raw values used to estimate the MGW at the central bp of a pentamer, based on both the unmethylated Pentamer Query Table (PQT) or its methylated version (*m*PQT). We used a nonparametric Wilcoxon-test to test our hypothesis.
Under the same hypothesis used for poly[$(A/T)_3$], PQT lacked occurrences of raw data for poly[$(A/T)_4$] with a CpG bp step at the flanks of the query pentamer. To address this issue, we performed additional all-atom MC simulations for the same set of unmethylated and methylated sequences. Paired *t*-test was used to perform hypothesis testing in this case.

DNase I cleavage data and statistical modeling

Data preprocessing

We used methylation status-dependent DNase I cleavage as model system to validate our high-throughput method *methyl*-DNAshape. DNase I is an endonuclease that cleaves the phosphodiester backbone of DNA [15,16]. In a genomic context, DNase I can be used to profile the accessible regions of chromatin in a process called "DNase I footprinting". DNase-seq is a sequencing-based method that utilizes DNase I cleavage to identify open regions of chromatin in a high-throughput manner. We used DNase-seq data generated from DNase I treatment in the IMR90 human cell line (GEO accession number: GSM723024). Data analysis revealed a sequence context-dependent bias of the DNase I cleavage activity. In particular, the presence of a methylated CpG step immediately downstream of the cleavage site resulted in a strong bias. We categorized each cleaved site as high or low methylation status, depending on the degree of methylation of CpG step(s) in the neighboring sequence. We used DNA methylation data generated by whole-genome shotgun bisulfite sequencing in the same cell line (GEO Accession ID: GSM432687-92) to determine high or low methylation status.

Analysis of the co-crystal structure of DNase I with DNA (PDB ID: 2DNJ) [17] revealed that positively charged arginine residues formed contacts in the minor groove immediately

upstream of the cleavage site. A larger fraction of variation in cut rates was explained by the sequence context 3-bp up- or downstream of the cleaved site, leading to a hexamer model of sequence- and methylation-status-dependent DNase I cleavage, as revealed in our previous study [15].

Results of the genome-wide analysis of phosphodiester cleavage events were recorded in tabular format. Based on the methylation level of the genomic region, five tables (tier 1 to tier 5, from lowest to highest level of methylation; available at https://doi.org/10.5281/zenodo.834334) were generated. Each table contains 4,096 hexamers with multiple entries depending on the frequency of cleavage. For example, the first hexamer entry in the tier 1 table consists of three rows (Additional file 9: **Table S4**). Information in the table can be summarized as follows: A total of 5664 phosphates of type AAApAAA in the genome that were cleaved once, and 7 that were cleaved twice. For example, the absolute phosphate cleavage count for AAApAAA equals $1 \times 5{,}664 + 2 \times 7 = 5{,}678$.

Following Lazarovici et al. [15], we normalized the absolute phosphate cleavage counts by the total counts of a given hexamer in the genome (Additional file 10: **Table S5**, column 4). Normalized values were further divided by the maximum relative phosphate cleavage rate (maximum value from column 4) to keep all values in the range [0, 1] (resulting in normalized values in column 5). These *Scaled Ratio* (*SR*; **Table S5**, column 5) values refer to relative cut rates of the most frequently cleaved hexamer (ACTpTAG). Absence of a CpG step in ACTpTAG leads to an unbiased comparison of *SR* values of unmethylated and methylated hexamers containing CpG step(s). *SR* values were converted into relative binding free energy ($\Delta\Delta G$) values by scaling to the negative log. The following equation represents the conversion process:

$$Relative\ Binding\ Free\ Energy\ (RBFE)_{\text{hexamer}} := \Delta\Delta G/RT_{\text{hexamer}} = -\log\ (SR_{\text{hexamer}})$$

Statistical modeling

To understand DNase I cleavage bias from a DNA shape perspective, we adopted a statistical modeling method, L1- and L2-regularized multiple linear regression, to refine our previously published shape-to-affinity model [16]. To build the predictive model, we only used unmethylated hexamer data, namely DNA shape features as predictors and *RBFE* values as response variables. DNA shape features of unmethylated hexamers were predicted using DNAshape [18].

Predictions from DNAshape are unavailable in flanking regions (Additional file 6: **Fig. S2**). To assign values in these regions in an unbiased manner, we extended the sequence flanks by a general nucleotide "N" (with N ∈ {A, C, G, T}) to create a pentamer window with the bp of interest at the center. For the leftmost or rightmost bp, we extended the window by two Ns. For the second bp from either the left or right flank, we extended the window by a single N. DNAshape values obtained for all possible permutations of pentamers formed by N (4 for single N, 16 for NN) were averaged to assign a single value at each position of the flanking regions.

Considering the very low count of observed cut events (Additional file 9: **Table S4**, column 2) relative to the number of available genomic positions (column 3), we concluded that the DNase I cleavage activity followed a Poisson process. To avoid uncertainties in counting, we used the following criteria:

$$\sigma \text{ or } S \leq 0.2 \times Observed\ Count_{hexamer} \ \ldots (1)$$

Where:

σ: standard deviation, S: sampling error

In a Poisson distribution, we can use standard deviation as an estimation of the sampling error. For a Poisson distribution:

$$\sigma = \sqrt{Observed\ Count_{hexamer}} \ \ldots (2)$$

Solving Eq. (1) using (2) gives us:

$$Observed\ Count_{hexamer} \geq 25$$

With the above considerations, we included the 3,037 hexamers with an absolute phosphate cleavage count ≥ 25 in the training set for the model (see ***Data Preprocessing*** for details). Because the model is linear, we can infer changes in *RBFE* (*ΔΔΔG*) by using these counts and the methylation-induced changes in shape features (*Δshape*).

$$\Delta\Delta\hat{G}_{methylated} = W^T Shape_{methylated} + b$$
$$\Delta\Delta\hat{G}_{unmethylated} = W^T Shape_{unmethylated} + b$$
$$\Delta\Delta\Delta\hat{G} = \Delta\Delta\hat{G}_{methylated} - \Delta\Delta\hat{G}_{unmethylated} = W^T \Delta shape$$

For modeling, we used the widely used tool *glmnet* with hybrid regularization (both L1- and L2-regularization by setting *alpha* = 0.5). Vignettes for *glmnet* are available at https://cran.r-project.org/web/packages/glmnet/vignettes/glmnet_beta.html.

CpG context for unmethylated DNA

Both DNAshape [18] and *methyl*-DNAshape (this work) are pentamer sliding-window based DNA shape feature prediction methods. In addition to offering the shape feature prediction, *methyl*-DNAshape offers users the ability to predict methylation-induced shape changes (*Δshape*; **Fig. 2**). However, simply subtracting the DNAshape feature vector from the *methyl*-DNAshape feature vector may not result in the *Δshape* originating solely from DNA methylation in all cases. For example, in the *m*PQT, the estimated MGW at the central A for pentamer 5′-TGATm-3′ is 5.23 Å, calculated by averaging the MGW values of all pentamers of this type in the methylated sequence pool. However, this pentamer always had a "g" (guanine following the methylated cytosine indicated by "m") at the sixth position flanking the pentamer on the 3' side, due to the assumption of mpg dinucleotide steps in case of methylated cytosines. The unmethylated counterpart of this pentamer, 5′-TGATC-3′, with estimated MGW value 4.77 Å, is averaged over any nucleotide (N ∈ {A, C, G, T}) at the sixth position flanking the pentamer in the

unmethylated sequence pool. Hence, ΔMGW (5.23 Å – 4.77 Å = 0.46 Å) is confounding methylation and sequence effects because of the identity of the nucleotide at the sixth position.

To address this technical subtlety, we compiled an additional table, called the CpG context table. We illustrated the use of this table to predict the ΔMGW (Additional file 6: **Fig. S2**). Apart from existing MC simulation data used to build the PQT used in DNAshape, we ran additional MC simulations to enrich the count for such pentamers with the CG context in their flanks. With this new query table, we believe that we can look at the effect of methylation on shape feature values more closely ($\Delta MGW_{DNAshape}$ = 0.46 *vs.* $\Delta MGW_{CpG\ context\ table}$ = 0.22 Å).

T-test for IUPAC-based shape analysis in Fig. 6

Two-tailed paired t-test statistics was used to infer the significance of ΔMGW for hexamers and pentamers of types NNAYCG or NGAYCG and NNACG or NGACG, respectively (**Fig. 6d**). The latter hexamers or pentamers where T replaces N at the initial position, representing the most preferred binding site (TGAYCG, count=2; TGACG, count=1), were not included in the plot because of too few possible instances to perform significance tests. Nevertheless, the ΔMGW for TGATCG is 0.22 Å, and for TGACCG it is 0.16 Å.

Validation of *methyl*-DNAshape using experimentally determined structures

Additional filtering, such as by experimental procedure (i.e., X-ray structures) and methylation status (i.e., fully and not hemi-methylated CpG steps), reduced the 20 structures (PDB IDs listed above) with methylated CpG step(s) to a total of only 10 structures. Such a very low count is obviously not sufficient for a comparative analysis or validation, especially given other influences such as crystal-packing artifacts. Nevertheless, we visualized the MGW profiles of some of these structures (Additional file 8: **Fig. S3**), and this limited comparison indicated agreement between the X-ray crystallography and *methyl*-DNAshape results. The limited experimental data do not provide validation, but rather emphasize the need for a computational method to fill the current gap in structural information on methylated DNA.

Additional references

[1] Berman HM. The Protein Data Bank. Nucleic Acids Res. 2000;28:235–42.
[2] Shannon P, & Richards M. MotifDb: An Annotated Collection of Protein-DNA Binding Sequence Motifs. R package 2017; version 1.20.0.
[3] Jolma A, Yan J, Whitington T, Toivonen J, Nitta KR, Rastas P, et al. DNA-binding specificities of human transcription factors. Cell 2013;152:327–39.
[4] Yang L, Orenstein Y, Jolma A, Yin Y, Taipale J, Shamir R, et al. Transcription factor family□specific DNA shape readout revealed by quantitative specificity models. Mol. Syst. Biol. 2017;13:910.
[5] Mathelier A, Fornes O, Arenillas DJ, Chen CY, Denay G, Lee J, et al. JASPAR 2016: A major expansion and update of the open-access database of transcription factor binding profiles. Nucleic Acids Res. 2016;44:D110–5.
[6] Wingender E. TRANSFAC: an integrated system for gene expression regulation. Nucleic Acids Res. 2000;28:316–9.

[7] Sklenar H, Wüstner D, & Rohs R. Using internal and collective variables in Monte Carlo simulations of nucleic acid structures: Chain breakage/closure algorithm and associated Jacobians. J. Comput. Chem. 2006;27:309–15.
[8] Rohs R, Sklenar H, & Shakked Z. Structural and energetic origins of sequence-specific DNA bending: Monte Carlo simulations of papillomavirus E2-DNA binding sites. Structure 2005;13:1499–509.
[9] Cornell WD, Cieplak P, Bayly CI, Gould IR, Merz KM, Ferguson DM, et al. A second generation force field for the simulation of proteins, nucleic acids, and organic molecules. J. Am. Chem. Soc. 1995;117:5179–97.
[10] Aduri R, Psciuk BT, Saro P, Taniga H, Schlegel HB, & SantaLucia J. AMBER Force Field Parameters for the Naturally Occurring Modified Nucleosides in RNA. J. Chem. Theory Comput. 2007;3:1464–75.
[11] Lavery R, Zakrzewska K, & Sklenar H. JUMNA (junction minimisation of nucleic acids). Comput. Phys. Commun. 1995;91:135–58.
[12] Lavery R, & Sklenar H. Defining the structure of irregular nucleic acids: conventions and principles. J. Biomol. Struct. Dyn. 1989;6:655–67.
[13] Rohs R, Jin X, West SM, Joshi R, Honig B, & Mann RS. Origins of specificity in protein-DNA recognition. Annu. Rev. Biochem. 2010;79:233–69.
[14] Rohs R, West SM, Sosinsky A, Liu P, Mann RS, & Honig B. The role of DNA shape in protein-DNA recognition. Nature 2009;461:1248–53.
[15] Lazarovici A, Zhou T, Shafer A, Dantas Machado AC, Riley TR, Sandstrom R, et al. Probing DNA shape and methylation state on a genomic scale with DNase I. Proc. Natl. Acad. Sci. U. S. A. 2013;110:6376–81.
[16] Dantas Machado AC, Zhou T, Rao S, Goel P, Rastogi C, Lazarovici A, et al. Evolving insights on how cytosine methylation affects protein-DNA binding. Brief. Funct. Genomics 2015;14:61–73.
[17] Lahm A, & Suck D. DNase I-induced DNA conformation. 2 Å Structure of a DNase I-octamer complex. J. Mol. Biol. 1991;222:645–67.
[18] Zhou T, Yang L, Lu Y, Dror I, Dantas Machado AC, Ghane T, et al. DNAshape: a method for the high-throughput prediction of DNA structural features on a genomic scale. Nucleic Acids Res. 2013;41:56–62.
