# Supplementary material for: Systematic prediction of DNA shape changes due to CpG methylation explains epigenetic effects on protein–DNA binding
Source: Epigenetics Chromatin. 2018 Feb 6;11:6. doi: 10.1186/s13072-018-0174-4 (PMC5800008; doi:10.1186/s13072-018-0174-4)
Supplement: Supplementary file 2 — Additional file 2. Table S1. Types of DNA fragments and their counts considered in MC simulations. [file 13072_2018_174_MOESM2_ESM.pdf]

Table S1. Types of DNA fragments and their counts. Summary of types of sequences considered for all-atom Monte Carlo (MC) simulations. Most sequences were designed to cover different flanking sequences. Sequences between “-” symbols in column 1 are “core sequences”. Other sequences are regarded as flanks. “N” in designed sequences represents general DNA alphabet letters {A, C, G, T}. Methylated cytosine (“m”) and subsequent guanine (“g”) bases are underlined.

Fragments	Number of MC simulations	Selected from
Human HOXA9 binding sequences	3	[1]
Human HOXA5 binding sequences	84	[1]
CGNN-5mer-NNCG	1054	Designed
CGNN-NNNNmg-NNCG	1298	Designed
CGCG-NNmgNN-CGCG	253	Designed
CGCG-NNNmgN-CGCG	256	Designed
CGNN-mgNNNmg-NNCG	496	Designed
CGNN-poly[A/T]$_4$mg-NNCG	74	Designed
**Total**	**3518**	

[1] Hu S, Wan J, Su Y, Song Q, Zeng Y, Nguyen HN, et al. DNA methylation presents distinct binding sites for human transcription factors. eLife 2013;2:e00726.
