# Supplementary material for: Systematic prediction of DNA shape changes due to CpG methylation explains epigenetic effects on protein–DNA binding
Source: Epigenetics Chromatin. 2018 Feb 6;11:6. doi: 10.1186/s13072-018-0174-4 (PMC5800008; doi:10.1186/s13072-018-0174-4)
Supplement: Supplementary file 3 — Additional file 3: Table S2. Variables considered in MC simulations. [file 13072_2018_174_MOESM3_ESM.pdf]

Table S2. Variables considered in MC simulations.

Type of variable	Count	Description
Collective	6	3 rigid-body rotations, 3 rigid-body translations of nucleotides
Internal	6 (7 for T or m)	Glycosidic torsion angle, two endocyclic torsion and one bond angle, sugar phase and amplitude (methyl group rotation for T or m)
