# Supplementary material for: Systematic prediction of DNA shape changes due to CpG methylation explains epigenetic effects on protein–DNA binding
Source: Epigenetics Chromatin. 2018 Feb 6;11:6. doi: 10.1186/s13072-018-0174-4 (PMC5800008; doi:10.1186/s13072-018-0174-4)
Supplement: Supplementary file 4 — Additional file 4: Figure S1. Shape vector calculation. [file 13072_2018_174_MOESM4_ESM.pdf]

a

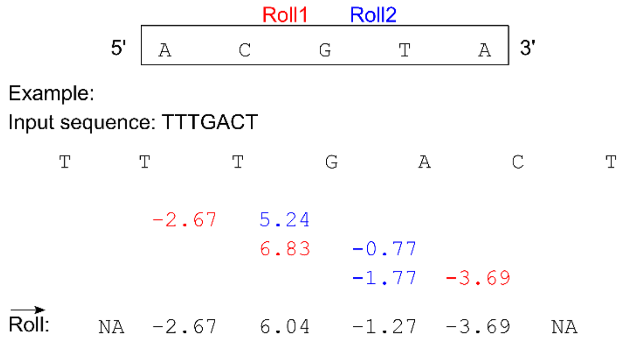

QueryTable entries:

Pentamer	<Roll1>	σ_{Roll1}	#Pentamer	<Roll2>	σ_{Roll2}	#Pentamer
TTTGA	-2.67	1.10	79	5.24	1.58	79
TTGAC	6.83	1.23	68	-0.77	0.87	65
AGTCA	-3.69	2.08	78	-1.77	1.41	78

b

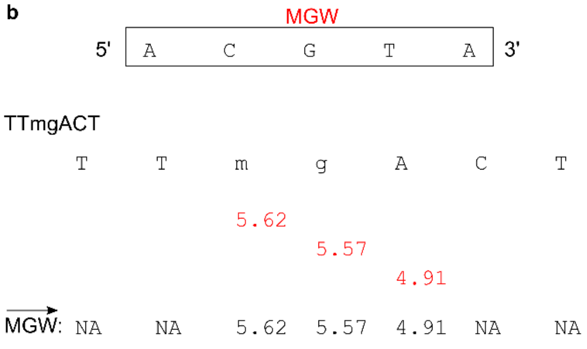

QueryTable entries:

Pentamer	<MGW>	σ_{MGW}	#Pentamer
TTmgA	5.62	0.17	141
GTmgA	5.57	0.14	152
AGTmg	4.91	0.19	78

Fig. S1. Shape vector calculation. a Two Roll values, Roll1 and Roll2, were assigned to a given pentamer by using the query table for bp steps 2-3 and 3-4, respectively (illustrated at the top). The PQT lookup procedure is explained for calculation of the Roll feature vector for DNA sequence 5′-TTTGACT-3′ as an example. Retrieval of the Roll feature vector for this sequence queries the lookup table three times for listed pentamers in the table. Because the third query pentamer, 5′-TGACT-3′, finds its reverse complement 5′-AGTCA-3′ in the table, the search resulted in the reversal of Roll1 and Roll2 values of 5′-AGTCA-3′. The same process was adopted for the base-pair step feature HelT. **b** Illustration of MGW feature vector calculation. The process is simplified in this case because the search returns a single value at the central bp for a given pentamer. MGW values for two flanking nucleotides are undefined because values at these positions cannot be calculated as per definition of minor groove.

< . >: average; σ: standard deviation.
