# Supplementary material for: Systematic prediction of DNA shape changes due to CpG methylation explains epigenetic effects on protein–DNA binding
Source: Epigenetics Chromatin. 2018 Feb 6;11:6. doi: 10.1186/s13072-018-0174-4 (PMC5800008; doi:10.1186/s13072-018-0174-4)
Supplement: Supplementary file 5 — Additional file 5: Table S3. Count breakdown of unique pentamer entries in methyl-DNAshape Pentamer Query Table (mPQT). [file 13072_2018_174_MOESM5_ESM.pdf]

Table S3. Count breakdown of unique pentamer entries in *methyl*-DNAshape Pentamer Query Table (*m*PQT).

	Regular DNA alphabet $\sum$ = {A, C, G, T}		DNA alphabet with two additional letters $\sum$ = {A, C, G, T, m, g} m: 5mC; g: G base-paired to 5mC			
**Strand orientation**	**5-mers**	**Count**	**5-mers containing “mg”**	**Count**	**5-mers beginning with “g” or ending with “m”**	**Count**
Forward	NNANN	256	mgNNN	64	NNNNm	256
	NNCNN	256	NmgNN	64	mgNNm	16
			mgmgN	4	NmgNm	16
			*mgNmg	4	NNmgm	16
					mgmgm	1
					*gNNNm	64
					gmgNm	4
Reverse	NNTNN	256	NNNmg	64	gNNNN	256
	NNGNN	256	NNmgN	64	gNNmg	16
			Nmgmg	4	gNmgN	16
					gmgNN	16
					gmgmg	1
					gNmgm	4
Total		1024		268		682
Strand-specific total		512		134		341

Total count of pentamers used in the query table = 512 + 134 + 341 = 987

*Only counted in forward strand
