# Supplementary material for: Systematic prediction of DNA shape changes due to CpG methylation explains epigenetic effects on protein–DNA binding
Source: Epigenetics Chromatin. 2018 Feb 6;11:6. doi: 10.1186/s13072-018-0174-4 (PMC5800008; doi:10.1186/s13072-018-0174-4)
Supplement: Supplementary file 6 — Additional file 6: Figure S2. Use of CpG context table in ΔMGW prediction. [file 13072_2018_174_MOESM6_ESM.pdf]

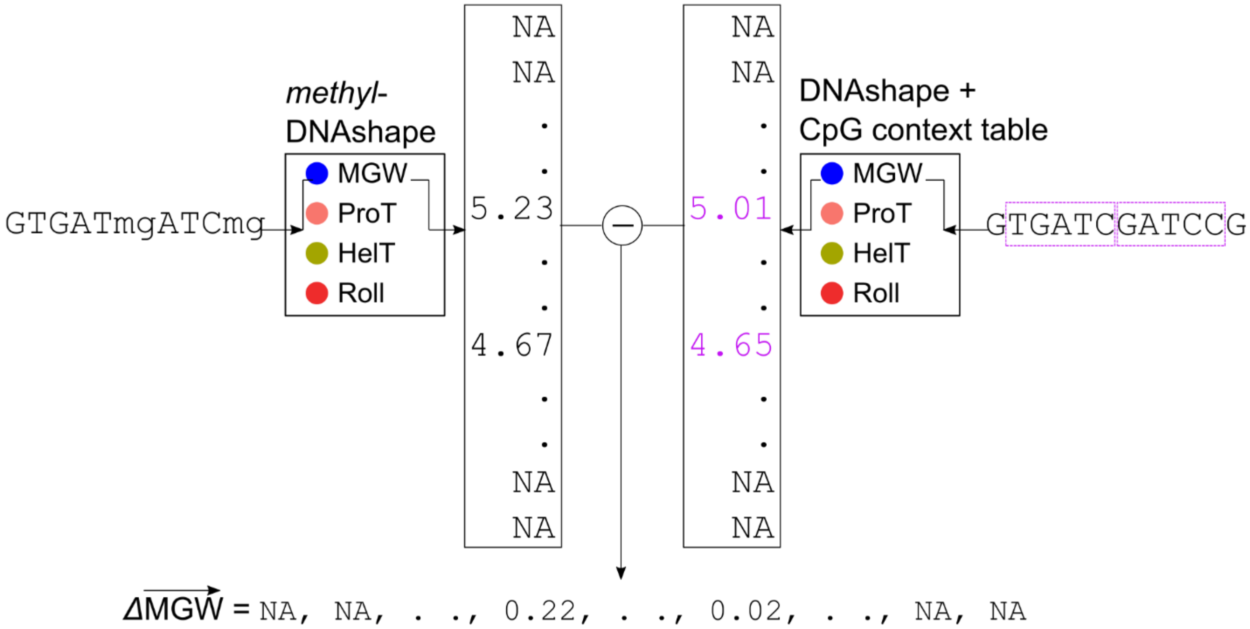

Fig. S2. Use of CpG context table in *Δ*MGW prediction. Example query sequence, 5′-GTGATmgATCmg-3′, used to illustrate *Δ*MGW prediction. Querying 'TGATm' (G<u>TGATm</u>gATCmg) to *methyl*-DNAshape gives MGW = 5.23 Å. Retrieving the value (5.01 Å) for its unmethylated version 'TGATC' (left magenta dotted rectangle) would require a lookup in the CpG context table. Similarly, the unmethylated version of pentamer 'gATCm' (GATCC; right magenta dotted rectangle) would require CpG contexts at both flanks. With compilation of the CpG context table, these specific cases can be taken into account.
