# Supplementary material for: Systematic prediction of DNA shape changes due to CpG methylation explains epigenetic effects on protein–DNA binding
Source: Epigenetics Chromatin. 2018 Feb 6;11:6. doi: 10.1186/s13072-018-0174-4 (PMC5800008; doi:10.1186/s13072-018-0174-4)
Supplement: Supplementary file 8 — Additional file 8: Figure S3. MGW profiles for selected DNA fragments or protein–DNA complexes. [file 13072_2018_174_MOESM8_ESM.pdf]

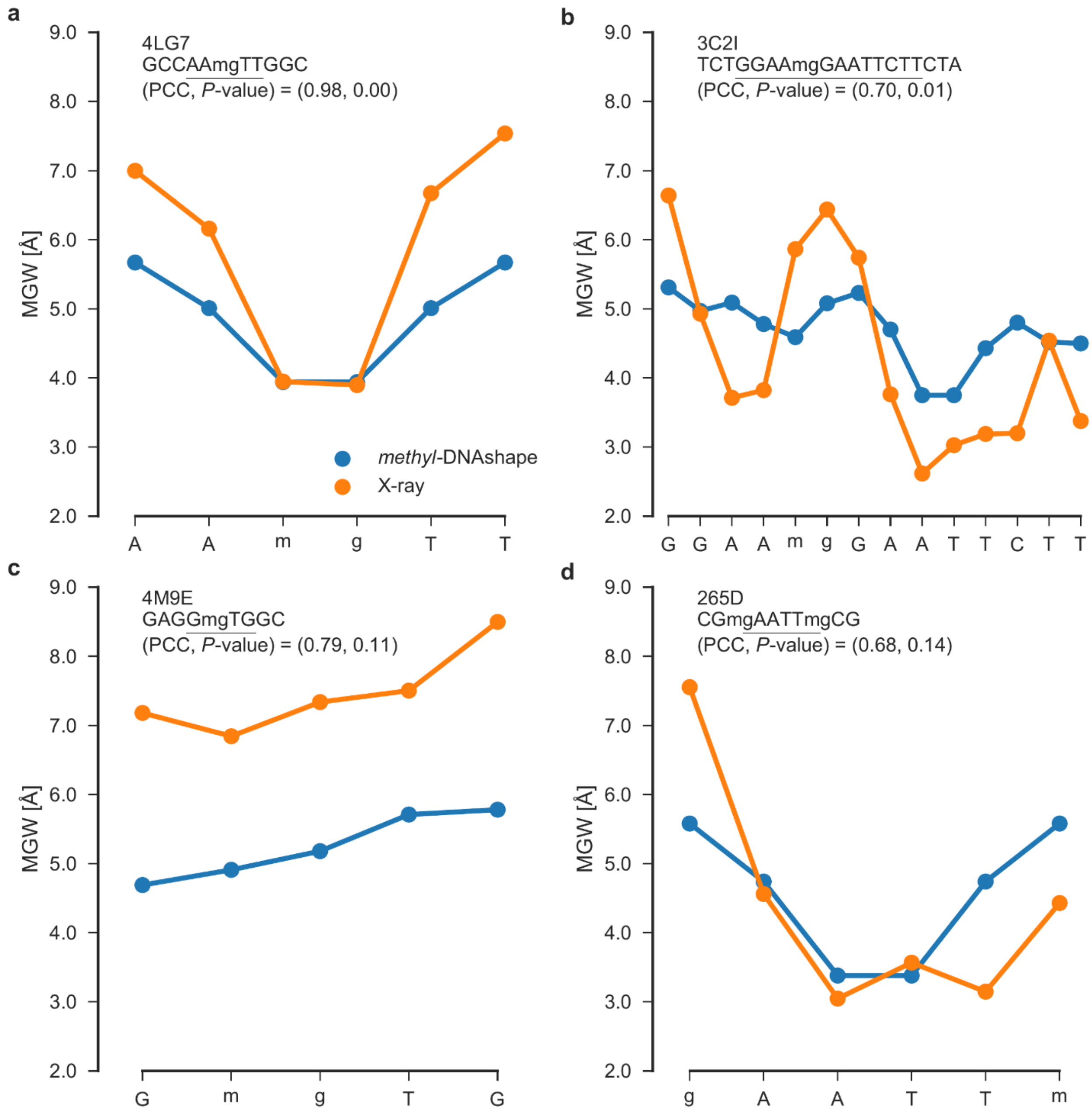

Fig. S3. MGW profiles for selected DNA fragments or protein-DNA complexes

a-d MGWs for DNA sequences (*x*-axis labels) of four structures (X-ray based; PDB IDs - 4LG7, 3C2I, 4M9E, and 265D) were predicted with *methyl*-DNAshape (blue points; this work) or calculated with CURVES (orange points; [1]). Underlined subsequence is expanded in every plot, presenting point-to-point correspondence between *methyl*-DNAshape predictions and CURVES-derived values of MGW. Pearson correlation coefficients (PCCs) between *methyl*-DNAshape and X-ray–based values and their corresponding *P*-values are included in each panel. Panels are shown in the order of significance (*P*-value).

[1] Lavery R, & Sklenar H. Defining the structure of irregular nucleic acids: conventions and principles. J. Biomol. Struct. Dyn. 1989;6:655–67.
