# Supplementary material for: Systematic prediction of DNA shape changes due to CpG methylation explains epigenetic effects on protein–DNA binding
Source: Epigenetics Chromatin. 2018 Feb 6;11:6. doi: 10.1186/s13072-018-0174-4 (PMC5800008; doi:10.1186/s13072-018-0174-4)
Supplement: Supplementary file 9 — Additional file 9: Table S4. Data preprocessing of DNase I cleavage data. [file 13072_2018_174_MOESM9_ESM.pdf]

Table S4. Data preprocessing of DNase I cleavage data. Entry of hexamer AAApAAA in tier1 table. Every table contains one or multiple entries of each hexamer.

Hexamer	Frequency	Count
AAApAAA	0	13037815
AAApAAA	1	5664
AAApAAA	2	7
