# Supplementary material for: Systematic prediction of DNA shape changes due to CpG methylation explains epigenetic effects on protein–DNA binding
Source: Epigenetics Chromatin. 2018 Feb 6;11:6. doi: 10.1186/s13072-018-0174-4 (PMC5800008; doi:10.1186/s13072-018-0174-4)
Supplement: Supplementary file 10 — Additional file 10: Table S5. DNase I cleavage data in hexamer context. [file 13072_2018_174_MOESM10_ESM.pdf]

Table S5. DNase I cleavage data in hexamer context. Observed Cuts (column 2) are counts of hexamers centered at cleaved phosphates (represented by "p"). Total number of mappable genomic positions for those hexamers is mentioned in column 3.

Hexamer	Observed Cuts	Genomic Positions	Ratio	Scaled Ratio
ACTpTAG	90,964	1,092,889	0.08323	1
ACTpTGT	99,223	1,284,748	0.07723	0.92790
ACTpTGG	91,281	1,360,831	0.06708	0.80590
ACTpTAA	119,341	1,840,040	0.06486	0.77924
TCTpTAG	85,512	1,335,788	0.06402	0.76912
...	...	...	...	...
CGGpTTT	10	201,805	0.00005	0.00060
CGCpGCG	3	81,371	0.00004	0.00044
GACpGCG	0	49,356	0	0

*Adapted from [2].

[2] Lazarovici A, Zhou T, Shafer A, Dantas Machado AC, Riley TR, Sandstrom R, et al. Probing DNA shape and methylation state on a genomic scale with DNase I. Proc. Natl. Acad. Sci. USA. 2013;110:6376–81.
